# Comparison of clinical outcomes in patients who underwent Gamma Knife radiosurgery for parasellar meningiomas with or without prior surgery

**DOI:** 10.1186/s12883-020-01731-2

**Published:** 2020-04-24

**Authors:** Yan-jia Hu, Yue-bing Xie, Li-feng Zhang, Chang Ding, Jing Chen

**Affiliations:** Department of Neurosurgery, West China Hospital of Sichuan University, Chengdu, Sichuan China

**Keywords:** Cavernous sinus, Parasellar meningiomas, Gamma knife radiosurgery, Surgery, Clinical outcomes

## Abstract

**Background:**

Parasellar meningioma is a common benign tumour in brain. Both surgery and radiosurgery are important treatment modalities for this tumour. The study was designed to investigate whether prior surgery would affect treatment outcomes of patients with parasellar meningiomas after management with Gamma Knife radiosurgery.

**Methods:**

A total of 93 patients who received Gamma Knife surgery were included in this retrospective study. There were 30 males and 63 females, with a median age of 48.6 years (range, 15.2–78.7 years). Prior surgery was performed in 45 patients. The median tumor volume was 5.02 cm^3^ (range 1.07–35.46 cm^3^) and median marginal dose was 12 Gy (range 10–15 Gy). The mean imaging follow-up and clinical follow-up periods were 40.7 and 52.7 months, respectively.

**Results:**

In the group without prior surgery, 31 patients had improvement of preexisting symptoms; and in the group with prior surgery, 20 patients were noted to improve. The difference in symptom improvement between the two groups reached statistical significance (*P* = 0.009). Patients with prior surgery were more likely to have stable symptoms after Gamma Knife surgery (*P* = 0.012). Tumor recurrence was reported in 8 patients out of 45 patients with prior surgery, and 3 patients out of 48 patents without prior surgery (*P* = 0.085). After Gamma Knife surgery, 5 and 4 patients in two groups developed new neurological symptoms, respectively (*P* = 0.651). Cox regression analysis identified follow-up period as prognostic factor of progression-free survival. Ordinal logistic regression analysis identified surgery prior to Gamma Knife surgery as an unfavorable factor of symptom change.

**Conclusion:**

Gamma Knife radiosurgery provided long-term effective tumor control and better symptom recovery compared with those with prior surgery. Patients with surgery before Gamma Knife radiosurgery were more likely to have stable symptoms. Further analyses indicated that long follow-up is essential to determine the efficacy of radiosurgery for parasellar meningiomas. Further study needs to include more patients with longer follow-up to draw a more solid conclusion.

## Background

Cavernous sinus is a very critical intracranial region encompassing many important neurovascular structures, such as oculomotor nerve, trochlear nerve, abducent nerve, and internal carotid artery. Meanwhile, various tumors originate from or invade cavernous sinus, causing cranial nerve dysfunction and compression of ICA. Parasellar meningiomas account for a vast proportion of these tumors and most of them are classified as WHO grade I [1]. Total surgical resection of parasellar meningioma is able to achieve favorable tumor control and even cure of tumors. However, surgical procedure of parasellar meningioma carries high risk of cranial nerve impairment and intraoperative bleeding [2, 3]. Although the advances in microsurgical and intra-operative monitoring techniques have improved surgical outcomes in recent years, surgical treatment of parasellar meningioma remains challenging [4–6]. According to a study of microsurgery for parasellar meningiomas, complete removal of tumor was achieved in 41.5% of patients (sample size 65); the incidence of new-onset cranial nerve deficit after operation was up to 54% (35 patients) [7].

As a radiosurgery procedure with minimal invasiveness, Gamma Knife radiosurgery (GKRS) is commonly used for skull base meningiomas [1, 8]. Since the first clinical article of GKRS for parasellar meningioma by Duma et al., [9] several studies employing GKRS for parasellar meningioma have been published, reporting 5-year progression-free survival (PFS) rates ranging from 80 to 99% and 10-year PFS ranging from 69 to 97% [10–19]. In addition to achieving comparable tumor control rates to microsurgical resection, GKRS appears to achieve a better neurological function preservation. The rate of neurological preservation is reported between 75 and 100% [20, 21].

Since the vast majority of parasellar meningiomas are histologically benign, tumor control as well as avoidance of cranial nerve dysfunction are both objectives of neurosurgeons. In the present study, we retrospectively analyzed the clinical outcomes in patients who underwent GKRS for parasellar meningioma with or without prior surgery to determine whether the previous surgery would change the treatment outcome of GKRS for parasellar meningioma.

## Methods

### Ethical approval

This study was approved by Biomedical Ethics Board of Sichuan University West China Hospital. Owing to the retrospective nature of the study, the requirement for informed consent was waived.

### Patient characteristics

We retrospectively reviewed Gamma Knife Plan System to identify patients harboring parasellar meningioma who underwent GKRS from January 2008 to September 2018 in West China Hospital affiliated to Sichuan University. Patients were included in this study if they met the following criteria: 1) with at least 6-month clinical and radiological follow-up; 2) single meningioma (patients with neurofibromatosis Type 2 were excluded); 3) patients diagnosed with WHO grade I meningioma after surgery; 4) MR imaging characteristics consistent with a benign meningioma in the parasellar neuroanatomical region preliminary for patients without prior surgery (e.g. dural tail sign, extraaxial location, uniform contrast enhancement, and even calcification).

A total of 93 patients were eligible for this study. There were 30 male and 63 female patients, with a median age of 48.6 years (range, 15.2–78.7 years). A total of 45 patients underwent surgical resection of parasellar meningioma prior to GKRS. At the time of GKRS, 14 patients had recurrent tumors and the other 31 patients had residual tumors. The median time between surgery and GKRS was 5.1 months (range 0.17–300 months). For patients with recurrent parasellar meningioma, the median interval between surgery and GKRS was 33.6 months (range 7–300 months). For patients with residual parasellar meningioma, the median interval between surgery and GKRS was 3.8 months (range 0.17–12 months). Tumor volume of patients ranged from 1.07 to 35.46 cm^3^ (median 5.02 cm^3^). Patient characteristics are detailed in Table 1. Comparison outcomes of patient characteristics between two groups are displayed in Table 2. Among the characteristics of patients to be compared, only age and follow-up period reached statistically significant difference. Patients without prior surgery were older and were more worried about potential risks of surgery. In a way, it is not surprising that the group without surgery had a shorter surveillance period prior to GKRS due to a greater awareness of GKRS as an available and effective treatment option. Other variates such as tumor volume, dosimetry and proportion of patients presenting with symptoms were comparable between two groups.

**Table 1:** Baseline characteristics of patients

Characteristics	Value
Sex	
Male	30 (32.3%)
Female	63 (67.7%)
Patients with prior surgery	45 (48.4%)
Median Age (range), yr	48.6 (15.2–78.7)
Median Tumor volume (range), cm^3^	5.02 (1.07–35.46)
Indication for GKRS	
Primary	48 (51.6%)
Residual	31 (33.3%)
Recurrent	14 (15.1%)
Median time interval between surgery and GKRS (range), month	
For all patients	5.1 (0.17–300)
For recurrent patients	33.6 (3.8–300)
For patients with residual tumor	3.8 (0.17–12)
GKRS parameters	
Median margin dose (range), Gy	12 (10–15)
Mean margin dose, Gy	12.2
Median maximum dose (range), Gy	25 (20–33.3)
Mean maximum dose, Gy	25.8
Median isodose line (range)	47% (43–50%)
Mean imaging follow-up in months (range)	40.7 (6–119.4)
Mean clinical follow-up in months (range)	52.7 (9.6–123.4)

*Abbreviations*: *yr* year; *GKRS* Gamma Knife radiosurgery

**Table 2:** Comparison of patient demographic between two groups

Variables	Patients with prior surgery	Patients without prior surgery	*P* values
Age (range), yr	48.1 (15.2–71)	55.2 (16.6–78.7)	0.004*
Follow-up, mo	47.0 (6.6–111.6)	34.8 (6–119.4)	0.037*
Tumor volume, cm^3^	8.77 (1.07–29.76)	7.78 (1.15–35.46)	0.500
Margin dose, Gy	12.2 (10–15)	12.3 (10–15)	0.681
Maximum dose, Gy	25.7 (20–31.1)	25.8 (22–33.3)	0.749
With symptoms, no. (%)	42 (93.3%)	41(85.4%)	0.319

*Abbreviations*: *yr* years, *mo* month, *no*. number; *, statistical significance. Continuous variables are described as means and ranges and categorical variables are described as numbers

A total of 83 patients presented with neurological symptoms at the time of GKRS. The most common neurological symptom was decreased visual acuity, followed by headache, facial numbness, and ocular motility disorders, et al. The most common cranial nerve deficit was optic neuropathy, followed by cranial nerves III, IV, VI deficits. Neurological symptoms pre-GKRS in 83 patients are illustrated in Table 3.

**Table 3:** Symptoms of each groups at the time of Gamma Knife Radiosurgery

Symptoms	With prior surgery	Without prior surgery
Headache	11	18
Facial numbness	5	11
Hearing loss	3	0
Ptosis	4	4
Ocular motility disorders	5	3
Visual field defect	5	3
Diplopia	2	5
Dizziness	1	0
Facial pain	0	4
Tinnitus	1	0
Facial paresthesia	2	2
Exophthalmos	1	2
Extremity numbness	0	3
Seizure	3	0
Facial paresis	2	0

### Radiosurgery technique

Radiosurgery was performed with a model C Leksell Gamma Knife. A Leksell stereotactic frame was fixed on the head of patient under conscious sedation and local anesthesia. After attachment of a fiducial system to the frame, all patients underwent either a high-definition computed tomography scan or volumetric MRI. Thin-sliced axial and/or coronal plane images were obtained after the administration of intravenous contrast when MRI was eligible for patients. When an MRI study could not be obtained because of medical contraindications (e.g., metallic material for surgical implants), a thin-slice stereotactic computed tomography was attained. All patients were discharged from the hospital within 24 h.

### Clinical and radiological assessment after GKRS

Patients were instructed to have clinical and imaging assessment at 6-month intervals in the first year and then yearly thereafter. Ophthalmological examination and audition test were performed when necessary. Meanwhile, patients were asked to make self-evaluation for their symptoms such as facial pain, facial numbness, paresthesia, and extremity numbness. Improvement of cranial nerve deficits was defined as improvement in function of at least 1 preexisting cranial dysfunction, and deterioration of cranial nerve deficits was defined as any aggravation of the preexisting cranial neuropathies. New-onset neurological symptoms was defined as occurrence of neuropathy after radiosurgery.

Tumor volumes at the time of GKRS were measured using Gamma Knife Plan System. Magnetic resonance images at follow-up were transported to Gamma Knife Plan System to measure tumor volume. Tumor regression was defined as tumor volume at least 50% lesser than the original volume; no change of tumor was defined as volumetric change within 25% of the original volume; and tumor progression (recurrence) was defined as tumor volume greater than 25% of the original volume. Tumor regression and stable status were viewed as tumor control.

### Statistical analysis

Data are presented as median or mean and range for continuous variables, and as frequency and percentage for categorical variables. Statistical analyses of continuous variables were performed using an unpaired Student t-test, regardless of difference in variance and Wilcoxon rank-sum tests when variables were classified as abnormal distribution. Statistical analyses of categorical variables were performed by means of chi-square or Fisher’s exact tests as appropriate. Kaplan-Meier plots of PFS were generated for the entire patients and patients in two groups, respectively. The comparison of PFS in two groups was conducted using log-rank test. A Cox proportional regression model was used to estimate the hazard ratio (HR) with its 95% confidence interval (CI) of all potential prognostic factors, including age, sex, follow-up, surgery prior to GKRS, marginal dose, maximal dose and et al. Factors with *p* < 0.15 in univariable analysis were entered into multivariable Cox regression analysis. Besides, multivariable logistic regression analysis was performed to identify factors predictive of unfavorable change in clinical presentation. Factor with *p* < 0.15 in univariable analysis was eligible for multivariable logistic regression analysis. All statistical analyses were performed using IBM SPSS (version 25.0; IBM Corp) software. A *p* value of < 0.05 was considered statistically significant.

## Results

### Clinical outcomes

At the time of GKRS, 42 patients who had prior surgery presented with symptoms. For patients without prior surgery, 41 patients presented with symptoms at GKRS. The incidences of symptoms did not show statistically significant difference between two groups. Clinical follow-up of 93 patients ranged from 9.6 to 123.4 months with the mean of 52.7 months. At the last follow-up, symptoms were improved in 51 patients, unchanged in 13 patients, and deteriorated in 19 patients. Control rate of preexisting symptoms was up to 77.1%. Overall, Mann-Whitney U test revealed that symptom changes were better in group without prior surgery (*P* = 0.009). Specifically, improvement rate of preexisting symptoms was higher in patients without prior surgery than those with prior surgery (P = 0.009); and stable symptoms were more likely in patients with prior surgery (*P* = 0.012). New-onset symptoms or signs were reported in 9 patients, and one of them was accompanied with tumor recurrence. In the group without prior surgery, 4 patients developed new symptoms; in the group with prior surgery, that number was 5. The incidence of new symptoms between two groups did not reach significant difference (*P* = 0.515). Memory decline harbored the highest incidence which accounted for 30% of the new symptoms, with all occurring in patients without prior surgery. Two patients who underwent prior surgery developed new-onset facial palsy. Given that the number of patients who developed new symptoms were very small, we did not compare the incidence of new-onset symptoms between two groups. The comparison of preexisting symptoms alteration between two groups as well the new-onset symptoms is displayed in Table 4.

**Table 4:** Changes of preexisting symptoms and the new-onset symptoms at the latest follow-up

	With prior surgery (*n* = 45), n (%)	Without prior surgery (*n* = 48), n (%)	*P* values
Patients with symptoms	42	41	0.319
Improved	20 (47.6%)	31 (75.6%)	0.009
Unchanged	10 (23.8%)	3 (7.3%)	0.012
Worse	12 (28.5%)	7 (17.1%)	0.213
New-onset symptoms	6 (13.3%)	4 (8.3%)	0.515

*GKRS* Gamma Knife radiosurgery. When we calculated the percentages of changes of preexisting symptoms, the number of patients with symptoms was used as denominator. Number of all patients was used as denominator when we calculated the percentages of new-onset symptoms in two groups

In order to predict the prognosis of preexisting symptoms, we performed ordinal logistic regression analysis. Surgery prior to GKRS (yes or no) was identified as the only prognostic factor of preexisting symptoms (OR = 2.99, 95% CI 1.31–6.83, *P* = 0.009) which would raise the risk of deterioration of preexisting symptoms.

### Radiological outcome

The mean imaging follow-up duration was 40.7 months (range, 6–119.4 months). At the latest follow-up, 60 patients (63.2%) showed no change in tumor volume, 24 patients (25.3%) had tumor regression, and 11 patients (11.6%) displayed tumor progression. The time interval between GKRS and diagnosis of recurrence ranged from 9.1 to 86.1 months (median 23.3 months). No change, regression, and progression of tumor volume were observed in 24, 14, and 7 patients in the group with prior surgery, respectively. In the group without prior surgery, no change, regression, and progression of tumor volume were observed in 36, 10, and 2 patients, respectively. No statistically significant difference between two groups regarding tumor response was obtained (*P* = 0.056).

**Fig. 1:**
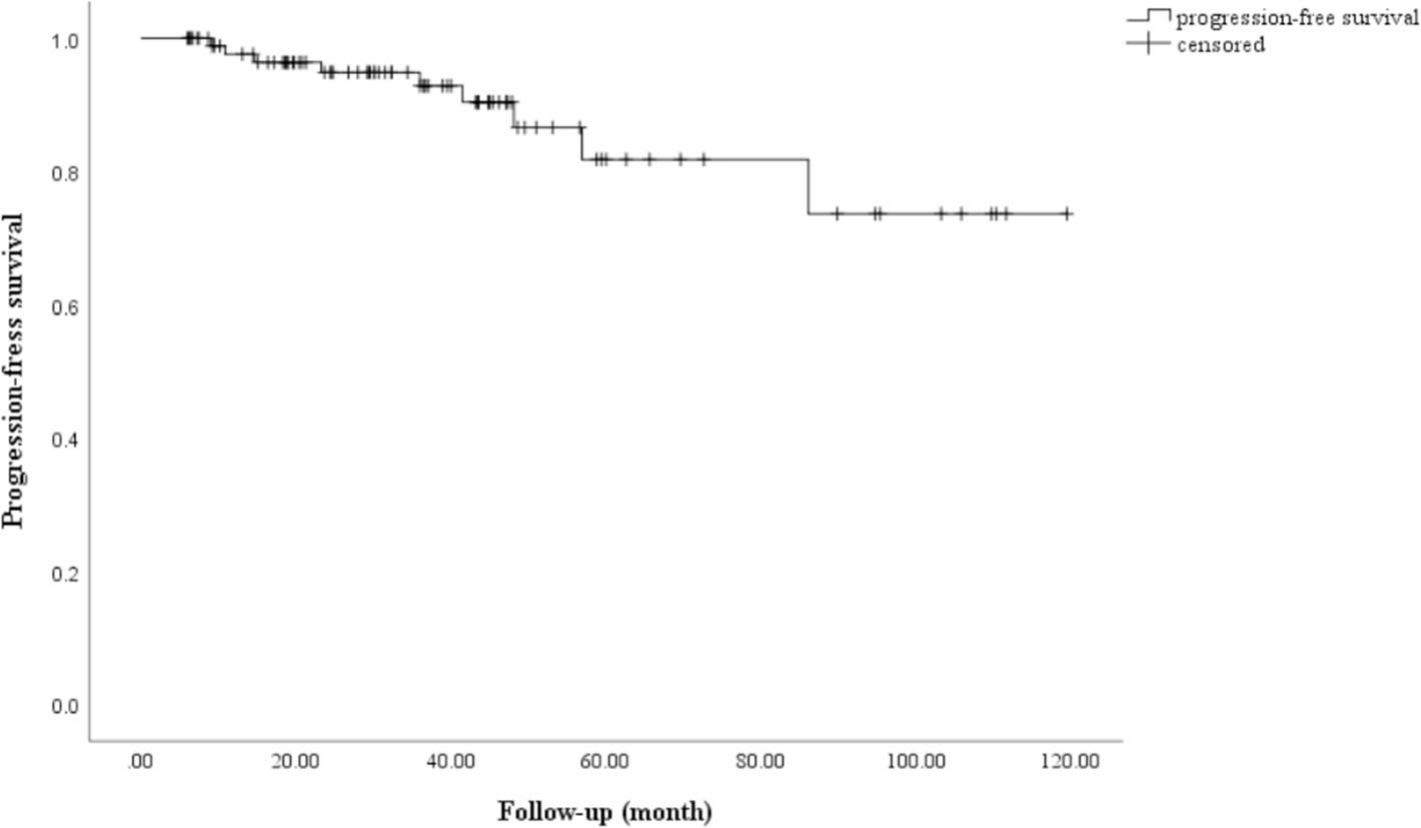
Kaplan-Meier analysis of progression-free survival after Gamma Knife radiosurgery (GKRS). The tumor control rate at 3, 5, 7 years after GKRS were 94.8, 90.4, and 73.6%, respectively

**Fig. 2:**
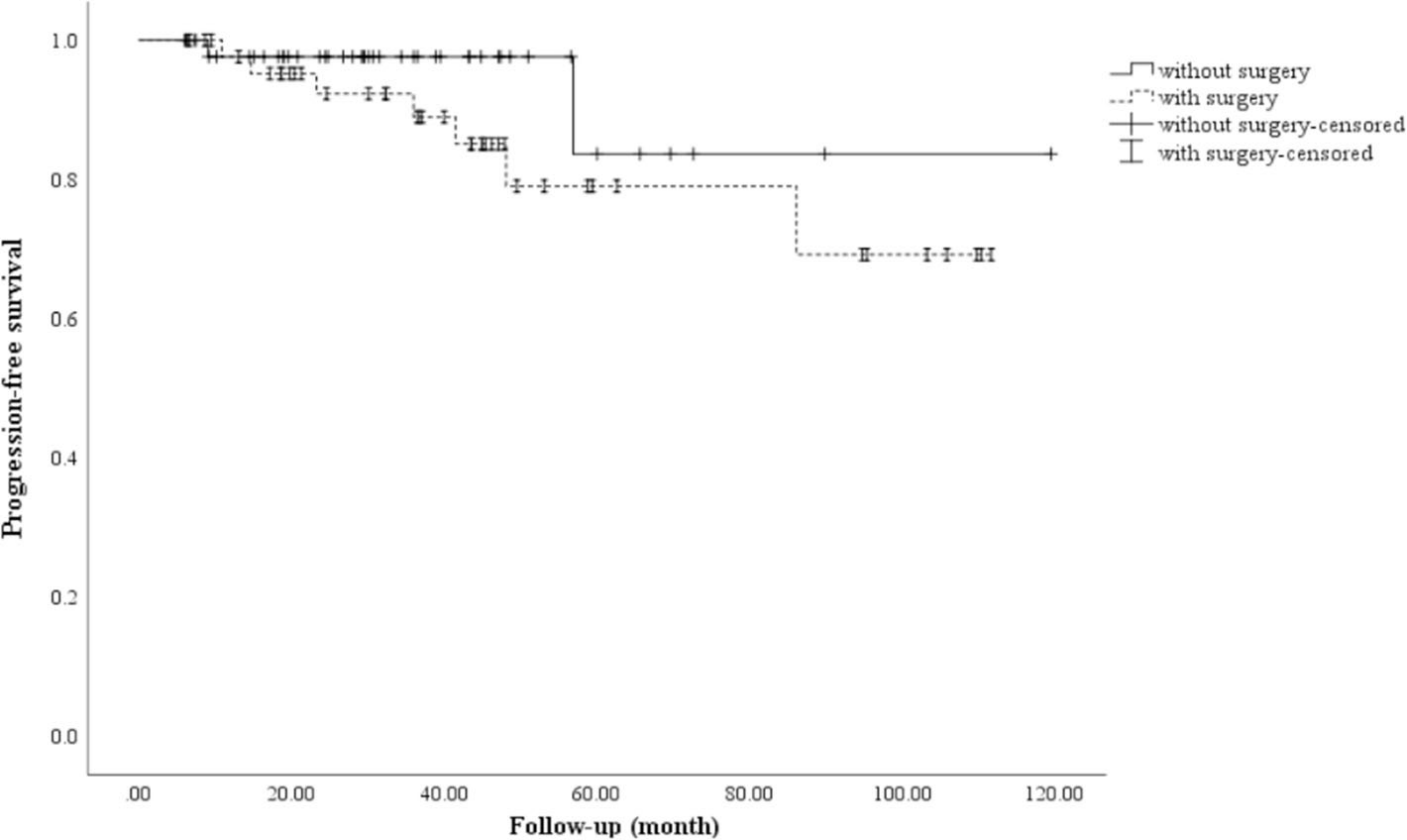
Kaplan-Meier analysis of progression-free survival after Gamma Knife radiosurgery (GKRS) regarding surgery prior to GKRS. Log-rank test showed no difference of PFS between two groups (*P* = 0.229)

Kaplan-Meier analysis demonstrated radiological PFS at 3, 5, and 7 years to be 94.8, 90.4, and 73.6%, respectively (Fig. 1). Univariate analysis only identified follow-up period as prognostic factor (follow-up period: HR = 0.939, 95% CI 0.884–0.998, *P* = 0.042). Prior surgery was not a prognostic factor of tumor control (*P* = 0.229, Fig. 2).

### Additional management

During follow-up, 11 patients had tumor recurrence (all with infield recurrence). Further treatments were as follows: 3 patients underwent repeat GKRS, 4 underwent craniotomy for tumor resection, and the remaining 4 continued to undergo observation every 6 months. Of the 4 patients who underwent observation, 2 died after tumor recurrence for 4 and 17 months, respectively. One of them died of meningitis and the other died of unknown reason.

## Discussion

In the present retrospective study, we summarized and analyzed clinical data of 95 patients harboring parasellar meningioma who underwent GKRS between January 2008 and September 2018 at West China Hospital. Patients were categorized into two groups in terms of surgery for parasellar meningioma prior to GKRS. We found that patients without prior surgery had better outcome of preexisting symptoms compared to those with prior surgery. We also observed that PFS of patients in two groups did not reach statistically significant difference, indicating that surgery prior to GKRS may not affect tumor control. In addition, we identified surgery prior to GKRS as a predictive factor for unfavorable outcomes of preexisting symptoms via multivariate Logistic Regression Analysis. We also found that tumor volume and follow-up duration were prognostic factors of tumor control via Cox Regression Analysis.

The cavernous sinus is composed of a large collection of thin walled veins on both sides of the sella, closely abutting to the optic chiasm and optic nerves [22]. In the cavernous sinus, neurovascular structures including ICA, oculomotor nerve, trochlear nerve, and abducens nerve are often invaded by parasellar meningioma, causing neurological deficits [23, 24]. Considering that the vast majority of parasellar meningioma are classified as WHO grade I, total resection of tumor can cure this extra-axial tumor. However, the complex structures within or adjacent to cavernous sinus make resection of parasellar meningioma challenging even with the advancement of microsurgical techniques over the last two decades. According to the published articles, the incidence of nerve dysfunction after microsurgery ranged from 17.9 to 74%, [5, 6, 25–27] and the recurrence rate of patients who underwent surgery as primary treatment ranged from 6.7 to 24.5% [25, 28]. In addition to surgery, stereotactic radiosurgery (SRS) is another option for the management of parasellar meningioma. Stereotactic radiosurgery has the advantages of precise dose administration and good preservation of peritumoral neurovascular structures [29, 30]. Over the years, an increasing number of studies have demonstrated the efficacy and safety of SRS for the treatment of parasellar meningioma. Importantly, in a meta-analysis including 17 series, Sughrue et al. [31] found that tumor recurrence of patients who underwent SRS alone was significantly lower than that of patients who underwent subtotal or total resection of tumor. Besides, they also found that the rate of postsurgical cranial neuropathy for patients who received surgery (59.6%; 95% CI, 7.4–16.1) was significantly higher than the rate of cranial neuropathy found in patients who underwent SRS alone (25.7%; 95% CI, 11.5–38.9%; *P* < 0.05). As a consequence, it seems that SRS is a superior treatment modality for parasellar meningioma than surgery regarding tumor control and post-treatment complications. In order to gain a better understanding of the management of parasellar meningioma, we conducted the present study to determine whether surgery prior to SRS would affect the change of preexisting symptoms and tumor control.

Symptom change is an important outcome in the clinical studies investigating parasellar meningioma because the vast majority of parasellar meningioma are WHO grade I and symptomatic. In the present study, symptom improvement was observed in 51 patients out of the 83 patients who presented with neurological symptoms before GKRS. Specifically, improvement rates of preexisting neurological symptoms of patients with or without previous surgery were 47.6 and 75.6%, respectively. The statistical analysis displayed that the difference of improvement rates of symptoms or signs between the two groups was significant (*P* = 0.009). Moreover, Cox proportionated-hazards analysis identified surgery prior to GKRS as a predictor for unfavorable outcome of symptoms. This result is consistent with other studies. Kano et al. [22] reported in their study that patients who had not undergone prior microsurgery had significantly higher improvement rates of preexisting cranial nerve symptoms (*P* = 0.01). They also reported that patients without prior microsurgery had a significantly higher likelihood of improvement of cranial nerve symptoms by Cox proportionated-hazards analysis (*P* = 0.001, HR, 3.06, 95% CI, 1.66–5.62). Furthermore, a systematic review including 49 articles by Lee et al. [31] also found that patients who had not undergone prior microsurgery had higher improvement rate of preexisting cranial nerve symptoms or signs (P = 0.001). Although this has been reported in previously articles, we additionally found that the whole outcomes of preexisting neurological symptoms were better in patients without prior surgery. However, this may be a “double-hit” phenomenon. We speculated that the preexisting symptoms may persist or deteriorate due to the injury during craniotomy. On the contrary, patients who underwent GKRS alone were more likely to experience improvement in symptoms or signs since these clinical presentations are attributable to tumor compression and cranial nerves involved do not suffer the potential injury by surgery. Once the tumor regresses, the symptoms or sign may be alleviated and even resolved. However, although improvement of symptom may be impaired by prior surgery, surgery plays an essential role in debulking or partially resecting large tumor (tumor diameter > 3 cm). Surgery can also provide the opportunity of GKRS for patients with parasellar meningioma.

Tumor control is another important outcome measure. In the present study, PFS was 94.8% at 3 years, 90.4% at 5 years and 73.6% at 7 years with a mean follow-up of 40.7 months. Prior studies indicated that the 3-year PFS after SRS ranged from 85 to 99% during follow-up intervals that ranged from 42 to 78 months [13, 15, 22, 32]. The 5-year PFS ranged from 80 to 99% during median follow-up intervals that ranged from 29 to 95 months. Furthermore, 7-year PFS after SRS ranged from 80 to 98% during follow-up intervals that ranged between 29 and 100 months [15, 33]. Among the 11 patients who experienced tumor relapse, 8 had undergone surgery prior to GKRS but the recurrence rates between two groups did not reach statistically significant difference. Of note, tumors of 6 patients relapsed within 2 years after GKRS, causing a lower tumor control rate. On the basis of Kaplan-Meier analysis, no significant difference in PFS was observed between two groups. In addition, multivariate analysis by Cox regression showed that surgery prior to GKRS was not a prognostic factor of tumor control. Accordingly, surgery prior to GKRS was not associated with either improvement or worsening of the PFS in the present study. Although the findings of our study concurred with that of Kano et al. [22], they are contrary to the findings of Sheehan et al. [34] who reported that prior surgery increased the risk of local relapse. The lack of consistency in this variable suggests further larger and longer follow-up studies need to be carried out to seek clarification although as the authors suggest there was a non-significant trend towards better control rates in patients without surgery.

In the present series, we identified follow-up duration as prognostic factor of tumor control by Cox multivariate regression analysis. Given the benign property of most parasellar meningioma, long-term follow-up is essential to obtain an accurate tumor control rate. Several studies reported that tumor recurrence occurred at 10 years or more after SRS [17, 35]. In our study, follow-up was predictive of better PFS. This phenomenon may be induced by the small patient population and selection bias. On the other hand, this phenomenon may suggest that meningiomas in cavernous sinus have a good prognosis with long-term follow-up. We did not find that tumor volume was predictive of bad tumor control. However, prior study indicated that tumor volume of > 14 cm^3^ was associated with tumor progression [36]. The reasons may be as follows: 1) they included a large patient population in their study; 2) the inclusion criterion was large skull base meningioma; and 3) the diagnostic criterion of tumor recurrence differed from ours (tumor greater than 15% of the original volume). To date, surgery should be considered first for patient with tumor diameter > 3 cm. Surgery can debulk or partially resect tumor to alleviate the compression of tumor to surrounding critical structures. Moreover, GKRS becomes feasible for those (patients with large tumors cannot be totally resected) who have already undergone surgery. In our study, a total of 48 patients underwent GKRS as the primary treatment for parasellar meningioma. Among them, tumor diameters of 44 patients were below 3 cm which is consider reasonable for this treatment. The other 4 patients chose GKRS instead of surgery, because some are in bad physical condition and some insist on GKRS.

In previously published series, incidence of cranial neuropathies after SRS ranges from 0 to 25% [14, 15, 25, 35, 37–39]. The incidence of new-onset neurological symptoms was 10.5% in our study and only one patient was relevant to tumor progression. Cranial neuropathies occurring after GKRS were observed in 6.3% of patients, indicating the safety of this treatment modality. In our study, difference in rate of new-onset neurological symptoms was not statistically significant, and we did not identify any other factors associated with that. Of note, according to the study by Williams et al., [11] factors associated with new deficits included larger tumor volumes (*P* = 0.05), lower margin doses (*P* = 0.004), tumor progression (*P* < 0.001), and longer follow-up duration (*P* = 0.03). Besides, three patients developed memory problems after GKRS. Large tumor and large radiation dose may result in memory problem after GKRS. However, in our study, tumor volumes and radiation doses of these three patients were within reasonable range. Thus, we were not sure whether this new-onset symptom was relevant to GKRS.

Our study has some limitations. The major limitation is the relatively small patient population and insufficiently long follow-up interval. In our institution, many patients harboring parasellar meningioma are ineligible in the present study because of loss to follow-up, preventing us from conducting a large series study. In addition, the diagnosis of meningioma by MRI may generate some bias. Our study is also limited by its retrospective nature and single-institution series. Finally, in this study we sought to determine the outcomes following planned single-session radiosurgery, and thus we did not include patients with multisession treatment plans or fractionated radiosurgery.

## Conclusion

Based on the present study, improvement in preexisting symptoms was more likely in patients who did not undergo prior surgery. Besides, patients with surgery before Gamma Knife radiosurgery were more likely to have stable symptoms. To date, there is no guideline for the management of parasellar meningioma; however, on the basis of the published studies, SRS is a suitable option for patients with relatively small tumor (i.e. diameter below 3 cm). For patients with larger tumors or severe symptoms, surgery can debulk or partially resect tumors and provides the opportunity for GKRS. Finally, considering the limitations of the present series, a multicenter study including more patients with long-term follow-up is warranted.

## Data Availability

The data and materials are available from the corresponding author if required.

## Abbreviations

GKRS: Gamma Knife radiosurgery
SRS: Stereotactic radiosurgery
PFS: Progression-free survival
HR: Hazard ratio
CI: Confidence interval
OR: Odds ratio
